# Spleen transcriptome response to infection with avian pathogenic *Escherichia coli *in broiler chickens

**DOI:** 10.1186/1471-2164-12-469

**Published:** 2011-09-27

**Authors:** Erin E Sandford, Megan Orr, Emma Balfanz, Nate Bowerman, Xianyao Li, Huaijun Zhou, Timothy J Johnson, Subhashinie Kariyawasam, Peng Liu, Lisa K Nolan, Susan J Lamont

**Affiliations:** 1 Department of Animal Science, Iowa State University, Ames, IA 50011, USA; 2 Department of Statistics, Iowa State University, Ames, IA 50011, USA; 3 Department of Poultry Science, Texas A&M University, College Station, TX 77843, USA; 4 Department of Veterinary and Biomedical Sciences, University of Minnesota, Saint Paul, MN 55108, USA; 5 Department of Veterinary and Biomedical Sciences, The Pennsylvania State University, University Park, PA 16082, USA; 6 Department of Veterinary Microbiology and Preventive Medicine, Iowa State University, Ames, IA 50011, USA

## Abstract

**Background:**

Avian pathogenic *Escherichia coli *(APEC) is detrimental to poultry health and its zoonotic potential is a food safety concern. Regulation of antimicrobials in food-production animals has put greater focus on enhancing host resistance to bacterial infections through genetics. To better define effective mechanism of host resistance, global gene expression in the spleen of chickens, harvested at two times post-infection (PI) with APEC, was measured using microarray technology, in a design that will enable investigation of effects of vaccination, challenge, and pathology level.

**Results:**

There were 1,101 genes significantly differentially expressed between severely infected and non-infected groups on day 1 PI and 1,723 on day 5 PI. Very little difference was seen between mildly infected and non-infected groups on either time point. Between birds exhibiting mild and severe pathology, there were 2 significantly differentially expressed genes on day 1 PI and 799 on day 5 PI. Groups with greater pathology had more genes with increased expression than decreased expression levels. Several predominate immune pathways, Toll-like receptor, Jak-STAT, and cytokine signaling, were represented between challenged and non-challenged groups. Vaccination had, surprisingly, no detectible effect on gene expression, although it significantly protected the birds from observable gross lesions. Functional characterization of significantly expressed genes revealed unique gene ontology classifications during each time point, with many unique to a particular treatment or class contrast.

**Conclusions:**

More severe pathology caused by APEC infection was associated with a high level of gene expression differences and increase in gene expression levels. Many of the significantly differentially expressed genes were unique to a particular treatment, pathology level or time point. The present study not only investigates the transcriptomic regulations of APEC infection, but also the degree of pathology associated with that infection. This study will allow for greater discovery into host mechanisms for disease resistance, providing targets for marker assisted selection and advanced drug development.

## Background

Maintaining proper food-animal health is important from an animal welfare, animal production and food safety standpoint. Avian pathogenic *Escherichia coli *(APEC) are a group of extraintestinal *E. coli *that commonly infect poultry. Infection can lead to colibacillosis, a disease that can be localized or systemic, with the more acute and serious forms ending in septicemia and death [1,2]. Colibacillosis is one of the most frequent diseases in poultry resulting in mortality losses at all stages of life and decreased production efficiency in older birds [1,3]. Mortality, decreased production and condemnation of contaminated product may result in significant economic losses to the poultry industry worldwide [1,2,4,5].

APEC can enter the food supply though contaminated eggs and meat [2,5,6], generating a path for human exposure. APEC has zoonotic potential, demonstrated by common virulence factors [7,8], genome similarities to human extraintestinal *E. coli *[9], and capacity to cause disease in animal models [10]. As such, APEC has been implicated as a possible source of *E. coli *responsible for urinary tract infections and meningitis in humans [8,11].

There is increasing pressure to reduce antimicrobial usage in livestock production. Other methods to maintain healthy, disease-free populations, such as enhanced host genetic resistance, have become a topic of great interest. Previous research has demonstrated the potential for genetic resistance to disease in poultry [12,13]. Greater understanding of host response to infection and resulting pathology will allow researchers to identify the genes that best convey protection.

The spleen is involved in both the humoral and cellular immune responses through its role in the generation, maturation and storage of lymphocytes [14,15]. Gene expression in the chicken spleen is commonly used as an indicator of immune response [16,17]. Here, we characterize the differences in splenic gene expression profiles between birds with mild and severe pathology, and of differing vaccination status over two time points in order to identify host traits that are associated with colibacillosis resistance.

## Methods

### Animals

In six replicates, 120 male non-vaccinated commercial broiler chicks were used at 1 day of age from a local hatchery (total n = 720). Birds were reared on wire-floored cages with *ad libitum *access to food and water, and a 22:2 hour light:dark cycle for the first 15 days and a 16:8 hour cycle thereafter. Half of the chicks were vaccinated with the increased serum survival protein (Iss) vaccine [18], given intramuscularly, at a dose of 0.5 ml/bird, containing 2 μg of vaccine and 50 μg of Quil A adjuvant in phosphate buffered saline (PBS) at 2 weeks of age. Non-vaccinated birds received 50 μg of Quil A adjuvant in 0.5 ml PBS at 2 weeks of age via the same route. The Iss vaccine is generated from a recombinant Iss protein fused to glutathione S-transferase (GST). *iss *is a gene encoding the increased serum survival outer membrane protein that is common in many APEC serotypes [19]. At 4 weeks of age, chicks were challenged with 0.1 ml containing 10^8 ^colony forming units of APEC O1, or PBS, by the intra-air sac route into the left thoracic air sac. Birds were euthanized and necropsied at 2 time points, 1 day and 5 days post-challenge, using half of the birds on each day. All animal research protocols were approved by the Iowa State University Institutional Animal Care and Use Committee.

### Lesion scoring

At necropsy, lesions were characterized and scores assigned for 3 internal tissues, air sacs, pericardium and liver, as described by Peighambari et al. [20]. Air sacs had a score range of 0 to 3, pericardium and liver had a score range of 0 to 2. A total lesion score was generated from the summation of lesion scores from each of the 3 tissues, with scores ranging from 0 to 7. Within each experimental replicate, the birds with the highest and lowest total lesion scores were designated as having mild or severe lesions. Birds with mild pathology had scores ranging from 0 to 2 with an average of 0.375 while those with severe pathology had scores ranging from 4 to 7 with an average of 6.125. Birds from the vaccinated, challenged group were not further subdivided by pathology and a bird with an average total lesion score for that day and experimental replicate were selected for analysis. Treatment groups are abbreviated by vaccination status (V for vaccinated or NV for non-vaccinated), challenge status (C for challenged or NC for non-challenged), day of necropsy (D1 for 1 day post challenge or D5 for 5 days post challenge), and, where utilized, pathology (M for mild or S for severe).

### Bacteria

APEC O1 strain (O1:K1:H7) was previously isolated from the lung of a turkey that died due to colisepticemia (NCBI Reference Sequence: NC_008563.1). It has been fully characterized and its genomic sequence is the only total APEC sequence presently in the public domain [9]. It was stored in Brain Heart Infusion (BHI) broth with 10% glycerol at -80°C. Two days prior to bacterial challenge, APEC O1 culture was streaked on Luria Bertani (LB) agar and incubated overnight at 37°C. After incubation, 10 ml of LB broth was inoculated with an isolated colony of APEC O1 from LB agar and incubated overnight at 37°C with shaking. On the day of challenge, the bacteria were pelleted by centrifugation at 5000 g for 15 minutes and the bacterial pellet washed 3 times with PBS. Bacteria were enumerated based on spectrometric reading at 600 nm, then the inoculum was adjusted to the desired bacterial concentration in PBS. Counts were confirmed through serial dilution plating of the inocula onto MacConkey agar overnight.

### Splenic RNA isolation

One sample from each of the 10 treatment groups, over 4 replicates, was selected for RNA isolation and microarray analysis. Spleen tissue was removed, diced and placed into 3.5 ml of RNAlater (Ambion, AM7021). Tissues were kept at 4°C for 7 days, then excess RNAlater was poured off, tissue transferred to 1.5 ml tube and stored in -80°C freezer. Isolation of RNA from spleen samples was performed using the Ambion MagMAX-96 for Microarrays Kit (AM1839) (Applied Biosystems, Foster City, CA). For each sample, 25 to 30 mg of tissue was placed into 600 μL of TRI Reagent Solution (Ambion). Tissues were then homogenized in the TRI Reagent. Following homogenization, 300 μL of homogenate was processed according to manufacturer's instructions, using the Spin Procedure. Total RNA was eluted using 50 μL of Elution Buffer and stored at -80°C. Quality and quantity of total RNA was assessed by Nanodrop (Thermo Scientific) and agarose gel electrophoresis. Across all 40 samples, the average 260/280 ratio was 2.06 with a standard deviation of 0.055. For 12 random samples, RNA Integrity Numbers were measured using Agilent Bioanalyzer 2100 (Agilent Technologies, Santa Clara, CA). An average RIN of 9.21 was achieved across all 12 samples.

### Microarray experiments

Four hundred nanograms of RNA was reverse transcribed into cDNA. During reverse transcription, a T7 promoter primer region was included into the cDNA. The cDNA was then transcribed back into cRNA, using T7 RNA polymerase, labeled with either Cy3 or Cy5 dye. The labeled cRNA was then purified using Qiagen RNeasy Mini Kit (Qiagen Inc., Valencia, CA). Labeled samples were assessed by Nanodrop for sufficient quantity and a minimum specificity of 8, where specificity is the concentration of the dye (pmol/μg) times 1000 divided by the concentration of the cRNA (ng/μL).

Each labeled dye (825 ng) was hybridized to Agilent 4 × 44 Chicken Microarray [21] for 17 hours at 65°C. Samples were arranged in a reference design, using the NV-NC-D1 sample from each experimental replicate as the reference to which all other samples within the replicate were hybridized. After hybridization, slides were washed using commercial Agilent Wash Buffer and Stabilization and Drying Buffer (Agilent Technologies, Santa Clara, CA) and scanned using GenePix 4100A scanner (Molecular Devices Inc., Sunnyvale, CA)

### Microarray analysis

Median signal intensities for each spot were background-corrected and log-transformed. The Locally Weighted Scatterplot Smoothing (LOWESS) procedure was used to correct the intensity-dependent dye bias for each 2-color array [22]. All technical control spots and any genes exhibiting an average signal to noise ratio (SNR) less than 3 over all 36 arrays were removed from analysis, where SNR is calculated as (median foreground - median background)/background SD for each dye. Likelihood ratio tests were conducted in R to determine the necessity of including random effects of array position, slide and experimental replicate in the model. The results of these tests showed no evidence of the presence of array position or slide effects and therefore only experimental replicate was included as a random effect. Treatment means were parameterized (Table 1) and estimated by fitting a linear mixed model to the difference of normalized signal intensities between the Cy3 and Cy5 channels for each array. The fixed effects of the linear mixed model include the effects of challenge, vaccination, severity (mild or severe), time point and interactions among them using the parameterization shown in Table 1. *P *values were obtained for all contrasts of interest and converted to q-values for false discovery rate control using the R package q-value that implements the method proposed by Storey and Tibshirani [23]. The data discussed in this publication have been deposited in NCBI's Gene Expression Omnibus (GEO) [24,25] and are accessible through GEO Platform accession number GPL6413 and Series accession number GSE25511 (http://www.ncbi.nlm.nih.gov/geo/query/acc.cgi?acc=GSE25511).

**Table 1:** Parameterization of treatment groups

Group	Parameterization
NV-NC-D1	μ

NV-NC-D5	μ + τ_1_

NV-C-D1-M	μ + β

NV-C-D5-M	μ + β + λ

NV-C-D1-S	μ + β + γ

NV-C-D5-S	μ + β + λ + γ + θ

V-NC-D1	μ + α

V-NC-D5	μ + α + τ_2_

V-C-D1	μ + α + β + φ

V-C-D5	μ + α + β + φ + τ_3_

Functional analysis of biological processes category was carried out using the Database for Annotation, Visualization and Integrated Discovery (DAVID) [26,27]. Lists of significant genes were analyzed against the background of all 24,851 genes included for further study.

### Quantitative PCR

Ten genes, *interleukin 1 beta, interleukin 6, avian beta-defensin 2, avian beta-defensin 6, avian beta-defensin 7, interferon gamma, toll-like receptor 2-type I, toll-like receptor 4, myeloid differentiation protein 2*, and *interleukin receptor 1-type II *were utilized to validate microarray results. Genes were selected based on their functions in immune response and significance within microarray results. Primer sequences are listed in Additional File 1. New primer sequences were designed using sequences from NCBI and PRIMER3 [28]. Individual spleen samples were run in triplicate, with each triplicate randomly distributed on the 96-well plate. RNA was quantified using QuantiTect SYBR Green kit (Qiagen Inc., Valencia, CA) as previously described by Redmond et al. [16]. Cycle threshold (Ct) values were recorded for each sample. Ct values were adjusted for starting concentration and reaction efficiency using the formula: 40 - (Sample Mean Ct Target + (Median 28S - Sample Mean 28S) * (Slope Target/Slope 28S)). Values were analyzed using the Fit Model procedure in JMP software (SAS Institute Inc., Cary, NC). Contrasts with significant differential expression in microarray analysis had fold change compared to qPCR results.

## Results

### Microarray

**Figure 1:**
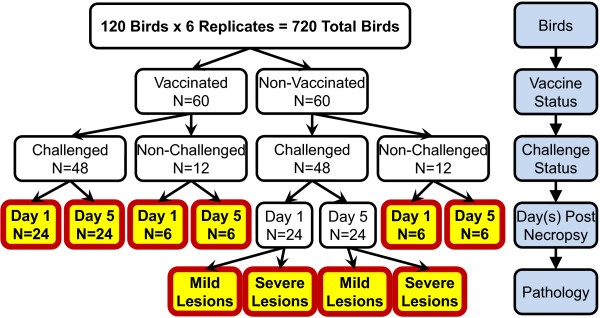
**Experimental design**. Flow chart of experimental design and 10 treatment groups showing how bird numbers within each replicate were placed into each of the 10 treatment groups.

**Figure 2:**
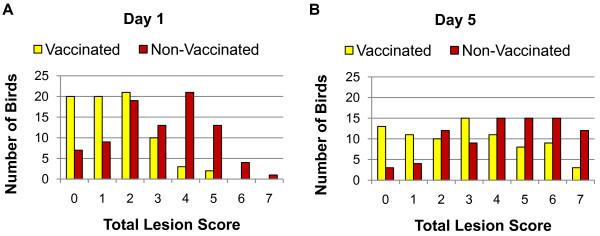
**Total lesion score distribution**. Comparison of the distribution of lesion scores of challenged birds taken on day 1 between birds that received the vaccine and birds that did not receive the vaccine for all 4 replicates on (a) day 1 and (b) day 5 (P < 0.001).

**Figure 3:**
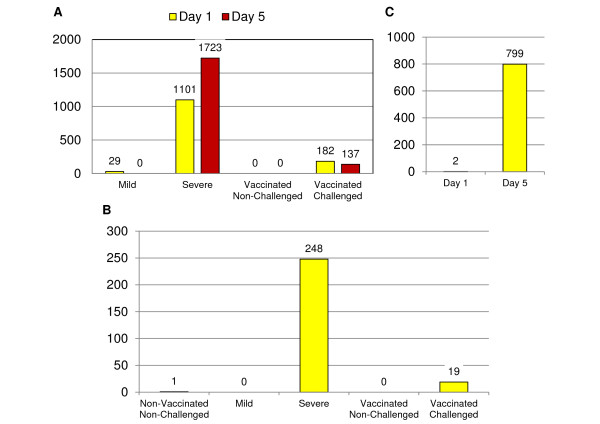
**Significantly differentially expressed genes for contrasts of interest**. Number of significantly differentially expressed genes between (a) treatment and control groups, (b) day 1 and day 5 within treatment, and (c) mild and severe groups (q-value < 0.05).

Ten groups were generated by dividing birds into vaccinated (V) and non-vaccinated (NV), challenged (C) and non-challenged (NC), sampled on 1 (D1) or 5 (D5) days post challenge, and the non-vaccinated challenged birds on both days were subdivided into mild (M) and severe (S) pathology (Figure 1). Birds were assigned to vaccination, challenge and time post-harvest groups *a priori*, and to pathology severity groups *a posteriori*. Samples from 40 individual birds (four biological replicates from each of 10 treatment groups) were used for gene expression study. After the removal of genes with low signal to noise ratio, 24,851 genes were included for further analysis. There was no detectible vaccination effect on spleen gene expression levels. Vaccination status had, however, a significant impact on total lesion score on day 1 (Figure 2a) and day 5 (Figure 2b) (*P *value < 0.001), reducing the total lesions observed in vaccinated birds. The number of significantly differentially expressed (DE) genes (q-value < 0.05) between treatment groups and NV-NC control groups were analyzed (Figure 3a). There were a large number of DE genes between NV-C severe and NV-NC control groups on day 1 (n = 1,101) and day 5 (n = 1,723). There were few DE genes between NV-C mild and NV-NC control groups on day 1 (n = 29) and (n = 0) on day 5. The change in gene expression over the two post-infection days was analyzed within each treatment group (Figure 3b). The NV-C severe group showed the most difference between the two days with 248 DE genes. The differences between the two pathologies, NV-C mild and NV-C severe, were analyzed (Figure 3c). More DE genes were seen on day 5 (n = 799) between pathologies than on day 1, (n = 2).

**Figure 4:**
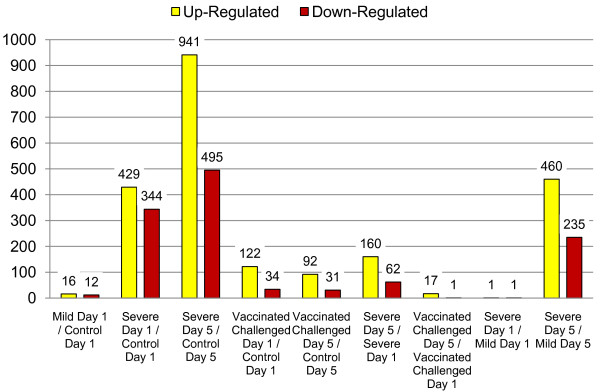
**Direction of response**. Directionality of significantly differentially expressed genes found in various contrasts with a minimum fold change of 1.5. For each contrast, up-regulated means there is greater expression in the first group, down-regulated means there is greater expression in the second group listed (q-value < 0.05).

**Figure 5:**
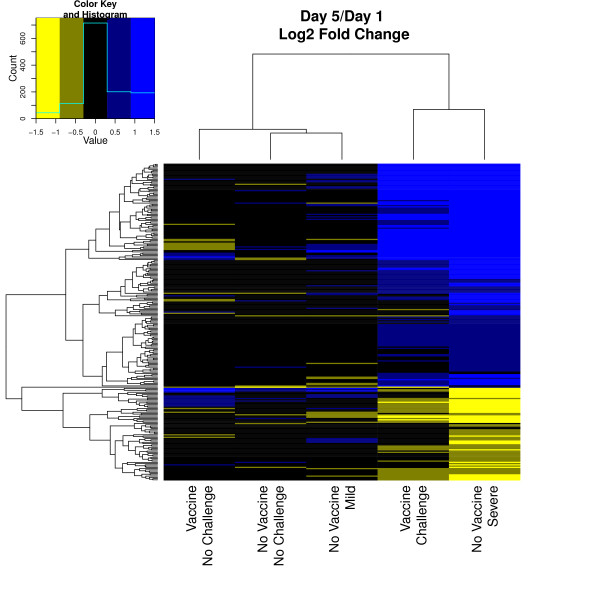
**Heatmap comparison of day 1 and day 5**. Visual representation of log2 fold change differences for all treatment groups between day 1 and day 5. Genes included were significant in at least one contrast presented (q-value < 0.05). Positive fold change indicates greater expression on day 5.

**Figure 6:**
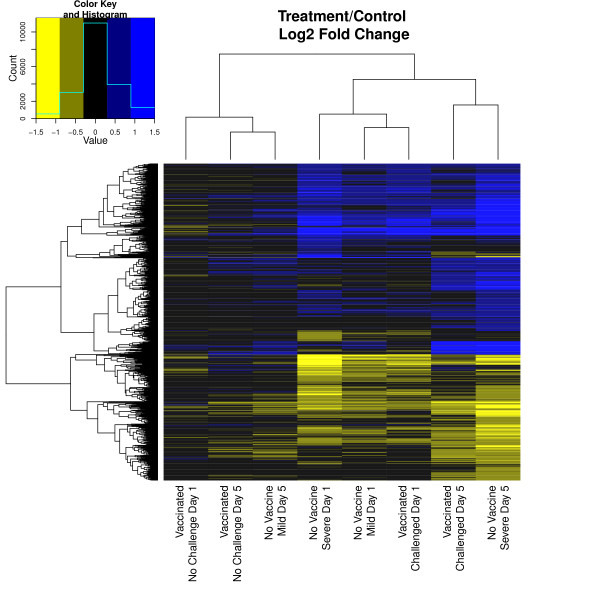
**Heatmap comparison of treatments and controls**. Visual representation of log2 fold change differences between all treatment groups and day appropriate NV-NC control. Genes included were significant in at least one contrast presented (q-value < 0.05). Positive fold change indicates greater expression in treatment group.

Direction and degree of difference between treatment groups was analyzed (Figure 4). Given a threshold of a minimum fold change of 1.5, there was a greater number of up-regulated genes due to increased pathology than the number of down-regulated genes. Visual comparison between multiple contrasts was generated. When comparing the change over time for all treatment groups, the NV-C mild pathology group clustered with both the NV-NC and V-NC groups; the NV-C severe pathology group and the V-C group clustered together (Figure 5). When comparing each treatment group to the NV-NC control group on each day, the NV-C mild group on day 5 clustered with the V-NC groups, while the NV-C mild group on day 1 clustered with the challenged groups (Figure 6).

### Gene ontology analysis

All contrasts in which there were sufficient DE genes (n > 29) were further analyzed for common biological processes (BP). For GO analysis, seven contrasts had significantly enriched BP terms. There were 156 unique BP terms, of which 95 were uniquely represented once across all 7 contrasts (See Additional Files 2, 3, 4, 5, 6, 7, 8). Many of the repeated biological process terms focused on white blood cell regulation along with defense/immune response to bacteria, and metabolic processes. The groups with the highest number of DE genes had the most biological terms common to these genes (Table 2). In contrasting NV-C severe and NV-NC control groups, on day 1 PI many biological processes were centered around immune and defense response, while day 5 PI heavily focused on regulation of white blood cells. The two most influenced KEGG pathways were the Jak-STAT signaling pathway and the cytokine-cytokine receptor interaction pathway, which occurred in the contrast between NC-C severe and NV-NC control on day 5 and in the contrast between NV-C severe and NV-C mild on day 5.

**Table 2:** Number of GO biological terms found in each contrast

Contrast	DE Genes	Biological Terms
NV-C Mild Day 1 vs. NV-NC Control Day 1	29	2

NV-C Severe Day 1 vs. NV-NC Control Day 1	1101	27

NV-C Severe Day 5 vs. NV-NC Control Day 5	1723	73

V-C Day 1 vs. NV-NC Control Day 1	182	27

V-C Day 5 vs. NV-NC Control Day 5	137	1

NV-C Severe Day 1 vs. NV-C Severe Day 5	248	4

NV-C Severe Day 5 vs. NV-C Mild Day 5	799	63

Number of differentially expressed (DE) genes found between contrasts of interest and the number of biological terms found using those genes.

### Quantitative PCR validation

Quantitative real time PCR (qPCR) was carried out on ten significantly DE genes, using a subset of the same RNA samples used for microarray analysis, to confirm the results seen on the microarray. Samples from vaccinated groups were not included in qPCR analysis due to no DE genes from vaccination effect, allowing analysis of 24 samples (4 replicates from 6 treatment groups). A normalizer gene, ribosomal 28S, was used to correct for starting template amount. Results from qPCR validation show general agreement with microarray results in direction of expression and significance (Table 3).

**Table 3:** Quantitative PCR validation

Gene	Contrast	qPCR	Microarray
AvBD 2	NV-C Severe Day 5 vs. NV-C Mild Day 5	-3.035*	-2.038**

	NV-C Severe Day 1 vs. NV-NC Control Day 1	2.954**	1.552**

	NV-C Severe Day 5 vs. NV-NC Control Day 5	-3.206**	-1.863**

	NV-C Severe Day 1 vs. NV-C Severe Day 5	5.525**	2.931**

AvBD 6	NV-C Severe Day 1 vs. NV-NC Control Day 1	2.592**	1.719**

	NV-C Severe Day 1 vs. NV-C Severe Day 5	4.726**	2.194**

AvBD 7	NV-C Severe Day 5 vs. NV-C Mild Day 5	-4.259	-3.440**

	NV-C Severe Day 1 vs. NV-NC Control Day 1	1.214	1.731**

	NV-C Severe Day 1 vs. NV-C Severe Day 5	4.446*	3.346**

IL1B	NV-C Mild Day 1 vs. NV-NC Control Day 1	0.567	3.826**

	NV-C Severe Day 1 vs. NV-NC Control Day 1	1.290	1.836**

	NV-C Severe Day 5 vs. NV-NC Control Day 5	2.296	1.960**

IL6	NV-C Severe Day 5 vs. NV-C Mild Day 5	2.263	1.117**

	NV-C Severe Day 1 vs. NV-NC Control Day 1	3.027*	1.227**

	NV-C Severe Day 5 vs. NV-NC Control Day 5	2.611	1.138**

IFNG	NV-C Severe Day 5 vs. NV-C Mild Day 5	2.399**	1.154**

	NV-C Severe Day 5 vs. NV-NC Control Day 5	2.427**	2.000**

TLR 2	NV-C Severe Day 1 vs. NV-NC Control Day 1	-0.070	1.140**

	NV-C Severe Day 5 vs. NV-NC Control Day 5	0.699	0.984**

TLR 4	NV-C Severe Day 5 vs. NV-NC Control Day 5	1.396*	1.336**

MD-2	NV-C Severe Day 5 vs. NV-C Mild Day 5	0.028	5.311**

	NV-C Severe Day 5 vs. NV-NC Control Day 5	0.034	1.230**

IL1-R, type II	NV-C Severe Day 1 vs. NV-C Mild Day 1	1.574 **	1.263**

	NV-C Severe Day 1 vs. NV-NC Control Day 1	1.241	2.676**

Log2 fold change between contrasts presented. + values indicate higher expression in the first group, - values indicate higher expression in the second group. ** *P *value < 0.05 in qPCR, q-value < 0.05 in microarray, * *P *value < 0.10 in qPCR

## Discussion

### Experimental design

This was a novel experimental design, allowing a contrast not only between challenged and non-challenged individuals, but also varying degrees of pathology. The large number of birds allotted into the challenged groups allowed the identification of a sufficient spread of lesion scores to separate pathology groups (Figure 1). These commercial birds were raised in a homogenous environment and exhibited a large spread in pathological response, suggesting that a mechanism other than environment, genetic variation, is responsible for the resistant or susceptible phenotypes and is available to select upon.

The common reference design was selected for this microarray experiment. Although there are loop designs that could gain some efficiency for some contrasts, the efficiency gain of could be eliminated in the instance of a loss of a microarray slide. This is because a rather big loop would be needed for our study of 10 treatment groups and the design of the microarray slide with 4 arrays per 1 slide would result in a large loss of data if a slide were to fail. However, reference design is very robust in this sense. Statistical calculation also shows that the reference design using the most naïve group, the NC-NV-D1 group, as the reference provides comparable variance estimates for our contrasts of interest with the loop design. The common reference design was suggested by Dobbin and Simon [29] because it produces better results than does a loop design for multiple comparisons and clustering analysis, both of which were applied in data analysis.

### Differential gene expression

Surprisingly, there was no significant vaccination effect on gene expression in the spleen. The vaccine, however, was clearly effective in protecting the chicks, as evidenced by the lesion scores. There are several variables that could contribute to no detection of DE genes in response to vaccination: statistical power, the tissue examined or the timeframe selected, in regard to age vaccinated, age challenged, and time points tissues were collected. Fold change calculations of these non-significant genes reveal few changes of 2 fold or greater, suggesting that inter-animal variation was not responsible for lack of detection of differential expression. The spleen is an important immunological organ in the chicken and has been successfully utilized to detect differences in cytokine gene expression after immunization Marek's disease vaccines and DNA vaccines [30-34]. The selection of vaccination at 2 weeks of age has also been successfully utilized to detect splenic expression differences [31], with several other vaccination times used successfully: day 18 of incubation, 1 day of age, and 2 weeks of age with a 4 week booster [30,32-34]. The time period selected between immunization and sampling can greatly impact the ability to detect expression differences, with later sampling times, 10 days post vaccination, showing reduced expression differences compared to earlier sampling times [31,33], though detection 26 days post vaccination is still possible [34]. It is likely that the selection of 1 and 5 days post challenge, 15 and 20 days after initial vaccination, was not optimal for expression discovery in the spleen utilizing an Iss vaccine.

There were more DE genes in the NV-C severe group than the V-C group on both days when contrasted against NV-NC control. Analysis of host-pathogen interaction genes [34] and cytokines [30] in response to Marek's Disease virus challenge revealed more DE genes in the spleens of non-vaccinated birds than vaccinated. This trend was again seen in birds challenged with *Salmonella *Enteritidis in the cecum of vaccinated and non-vaccinated birds [35]. Non-vaccinated birds that receive a pathogen challenge may rely more on significant changes in gene regulation to fight off bacteria than vaccinated birds. The greater amount of DE genes detected may also be attributed to the selection of average lesioned birds for representatives of the V-C group, though the number of DE genes is still a small fraction of that generated by the severe group.

Effect of APEC challenge on splenic gene expression varied with level of pathology. The contrasts in which there were the largest numbers of DE genes involved the NV-C severe group. Severe pathology may produce the largest changes in gene expression levels making them easier to detect, as observed with serum cytokine levels [36]. The NV-C mild group varied little from the NV-NC control, suggesting that the bacteria may have been cleared and the bird returned to homeostasis by day 5, while still exhibiting a small response on day 1. In contrast, the number of DE genes increased from day 1 to day 5 in the NV-C severe group compared to the NV-NC control. Past studies utilizing *Salmonella *and two distinct genetic populations illustrated differences in gene expression patterns between mild and severe pathologies unique to a genetic background [37], demonstrating the importance of assessing responses in lines of interest, such as a commercial line used in this study, for potential application. Commercial type birds, broiler chickens and broiler × Leghorn chickens, noted the same increase in gene expression over time under severe pathology, while chickens with a more robust chicken background, broiler × Fayoumi, had fewer DE genes that decreased over time under severe pathology [37]. Due to the clear difference in number of DE genes seen between either NV-C mild or NV-C severe and the NV-NC control group, it is unusual that there is not a higher number of DE genes detected between the NV-C mild and NV-C severe groups on day 1. Levels of serum amyloid A and cytokines showed a linear trend between control, mild, moderate and severe conditions [36]. This suggests that this is the trend for splenic gene expression level between NV-NC control, NV-C mild and NV-C severe groups. This intermediate expression could make it more difficult to detect expression differences, while the large expression level changes in the NV-C severe group make it easy to detect them against NV-NC control.

At both tissue harvest times, more genes increased expression in challenged groups compared to NV-NC control groups (55-78%) and more increased in severe compared to mild pathology group on day 5 (66%). Bacterial challenge has been demonstrated to cause more gene induction than repression [37], particularly within cytokines [38]. Direction stayed consistent for genes that were significant on both days. Of the genes that were differentially expressed in NV-C severe compared to NV-NC control on both days, 385 of 387 were in the same direction of regulation and all 6 DE genes on both days in the V-C groups were in the same direction of regulation.

In generation of the heatmaps, genes included were largely driven by the NV-C severe group, requiring a minimum q-value of 0.05 across all contrasts included. Comparing the change over time for each treatment group (Figure 5), groups clustered as expected based on earlier contrast comparisons. The NV-C mild pathology group showed little difference compared to NV-NC control and also showed more similar expression pattern changes to the V-NC group than to the NV-C severe and V-C group. As expected, both the NV-NC and the V-NC groups exhibit minimal changes of 2 fold or greater, further illustrating the lack of expression changes over time without a pathogen stimulus. When comparing each treatment with the NV-NC control (Figure 6), the NV-C mild group on day 1 was most similar to the other challenged groups, while on day 5 it clustered with non-challenged groups, supporting the hypothesis of returning to homeostasis, or a non-challenged state, by day 5. Contrasts that represented the same day post challenged clustered together, demonstrating strong similarities in expression changes over time among groups.

### Gene ontology investigation

The advantage of utilizing a global microarray is the potential to investigate and discover a wide range of gene ontology (GO) terms hidden within the dataset. Although it is difficult to differentiate cause and effect in gene expression in a pathogen challenge, functional terms enriched in the biological processes may grant some insights. Terms related to immune response, regulation of immune related cells, and metabolic processes commonly appeared in contrasts involving the NV-C severe group on both days or V-C group on day 1, consistent with bacterial infection as detected in multiple tissues [39-42].

The NV-C severe group on day 1 had many terms related to response to bacteria, inflammation and circulatory processes, along with a few receptor signaling terms. Particular genes among these groups included the avian beta-defensins, known peptides with antimicrobial activity that have demonstrated induction patterns in various tissues in response to *E.coli *derived lipopolysaccharide and *Salmonella *[43-45]. Toll-like receptors (TLR) recognize conserved molecular patterns common to many pathogens. Changes in TLR expression, in response to bacterial infection, have also been demonstrated [17,46]. Induction of pro-inflammatory response after pathogen challenge is common [47]. The changes seen in inflammation and circulatory processes may be responsible for observed lesion phenotype.

At day 5, many of the terms found in the NV-C severe group had changed, focusing more on regulation of white blood cells, localization and transport, with lesser emphasis on metabolic and biosynthetic processes than day 1. The severe status of these birds may be attributed to a slower response by these defense mechanisms. Cytokines and chemokines help signal white blood cells and attract them to sites of infection. Many cytokines have reported expression differences in less than 5 days post infection [42,48]. The main detectable differences between NV-C mild and NV-C severe groups on day 5 involved response mechanism and regulation of WBC. The Jak-STAT and cytokine-cytokine receptor interaction pathways have several overlapping elements, 6 and 9 overlapping elements in contrasts of NV-C severe vs. NV-C mild on day 5 and NV-C severe vs. NV-NC control on day 5 respectively. Bacterial infection has previously noted changes in the Jak-STAT pathway in granulosa cells [40] and cecal tissue [39]. Changes in genes in these pathways have also been discovered in spleen after *Clostridium *infection: signal transducer and activator of transcription, growth factor receptor-bound protein, cytokines and cytokine receptor genes [42].

The low number of DE genes found between several contrasts of interest limited GO analysis. Only one biological process term was significantly enriched in two contrasts examined: intracellular signaling cascade, between NV-C mild and NV-NC control groups on day 1 and transcription, between V-C and NV-NC control on day 5. As with many current microarrays, annotation has limited the extent of GO analysis, illustrating the urgent need to increase our knowledge in gene functions of genome sequences which have been discovered [49].

## Conclusions

There is a large difference in splenic transcriptome profiles between birds with mild and severe lesions in response to APEC infection, revealing gene networks potentially associated with disease resistance. The response of birds with severe lesions is much larger, both in magnitude and number of differentially expressed genes, than birds with mild lesions. Time post-challenge with APEC also resulted in significant differences in gene expression. Few differences were detected between the NV-C mild group and NV-NC control at day 1 and zero at day 5, suggesting that immune response to APEC was very rapid, occurring before day 1 sampling, or involved few detectable gene expression changes. Vaccination generated an efficacious protective effect, but no expression differences were detected at 2 weeks post vaccination. The gene ontology terms found within uniquely differentially expressed genes of birds with severe lesions helped provide insight into what genes are different as well as the overall processes defined by those genes. Changes in the Jak-STAT pathway and cytokine-cytokine receptor signaling highlight the importance of proper signaling cascades to fight infection. The results from this study add greater depth to the knowledge base about chicken host response to APEC.

## Authors' contributions

ES participated in necropsy, processed tissues, performed microarray experiments, analyzed data, conducted qPCR validation and drafted the manuscript. MO participated in the development of the microarray experimental design and assisted in data analysis. EB participated in qPCR validation. NB participated in qPCR validation. XL mentored and supervised microarray experiments. HZ provided equipment and annotation files for microarray experiments. TJ participated in the design of the study and necropsy. SK participated in the design of the study, purified Iss-GST fusion protein, and oversaw animal experiments (prepared Institutional Animal Care and Use Committee protocols and, performed vaccination, bacterial challenge and necropsy). PL participated in design of the study, necropsy and data analysis. LN participated in the design of the study, necropsy and oversaw animal experiments and bacterial challenge. SL participated in the design of the study, necropsy and interpretation of the results. All authors read, edited and approved the final manuscript.

## Supplementary Material

Additional file 1 **Primers utilized for qPCR analysis**. Forward and reverse primer sequences used for quantitative PCR analysis. Click here for file

Additional file 2 **Mild vs. Control, Day 1**. Results of DAVID analysis of significant genes using a custom background of genes included in the microarray analysis. Results presented are of the Biological Processes ALL category. Click here for file

Additional file 3 **Severe vs. Control, Day 1**. Results of DAVID analysis of significant genes using a custom background of genes included in the microarray analysis. Results presented are of the Biological Processes ALL category. Click here for file

Additional file 4 **Severe vs. Control, Day 5**. Results of DAVID analysis of significant genes using a custom background of genes included in the microarray analysis. Results presented are of the Biological Processes ALL category. Click here for file

Additional file 5 **Vaccinated Challenged vs. Control, Day 1**. Results of DAVID analysis of significant genes using a custom background of genes included in the microarray analysis. Results presented are of the Biological Processes ALL category. Click here for file

Additional file 6 **Vaccinated Challenged vs. Control, Day 5**. Results of DAVID analysis of significant genes using a custom background of genes included in the microarray analysis. Results presented are of the Biological Processes ALL category. Click here for file

Additional file 7 **Severe vs. Mild, Day 5**. Results of DAVID analysis of significant genes using a custom background of genes included in the microarray analysis. Results presented are of the Biological Processes ALL category. Click here for file

Additional file 8 **Severe Day 1 vs. Severe Day 5**. Results of DAVID analysis of significant genes using a custom background of genes included in the microarray analysis. Results presented are of the Biological Processes ALL category. Click here for file
