# Supplementary material for: Spleen transcriptome response to infection with avian pathogenic Escherichia coli in broiler chickens
Source: BMC Genomics. 2011 Sep 27;12:469. doi: 10.1186/1471-2164-12-469 (PMC3190404; doi:10.1186/1471-2164-12-469)
Supplement: Additional file 1 — Primers utilized for qPCR analysis. Forward and reverse primer sequences used for quantitative PCR analysis. [file 1471-2164-12-469-S1.DOC]

## Table 2 - Primers utilized for qPCR analysis

| Primer | Forward Sequence (5’-3’) | Reverse Sequence (5’-3’) | Reference |
| --- | --- | --- | --- |
| AvBD2 | TTTCTCCAGGGTTGTCTTCG | AGCAGCTTCCGACTTTGATT | Designed for Current Study |
| AvBD6 | TGCAGGTCAGCCCTACTTTT | GTCCACTGCCACATGATCC | Designed for Current Study |
| AvBD7 | CTGCTTTCCAGGGATCTGTC | GCCAGAGAAGCCATTTGGTA | Designed for Current Study |
| IL1β | GCTCTACATGTCGTGTGTGATGAG | TGTCGATGTCCCGCATGA | Withanage et al., 2004 Infection and Immunity, 72:2152-2159. |
| IL6 | GCTCGCCGGCTTCGA | GGTAGGTCTGAAAGGCGAACAG | Kaiser et al., 2000 Microbiology, 146:3217-3226. |
| IFNγ | GTGAAGAAGGTGAAAGATATCATGGA | GCTTTGCGCTGGATTCTCA | Kaiser et al., 2000 Microbiology, 146:3217-3226. |
| TLR2 | CCGGTGCTTCATTCACAGAT | CATATCCCATGCTCCTTTCC | Designed for Current Study |
| TLR4 | GGATCTTTCAAGGTGCCACA | CAAGTGTCCGATGGGTAGGT | Abasht et al., 2008 Veterinary Immunology and Immunopathology, 123:314-323 |
| MD2 | ATGAAGCATCCCACCCAATA | AATGAGCTGAAGAATCACAAAAAGT | Designed for Current Study |
| IL1R | TCCAGTGGTACAAGGATGCTC | GCCGGAGTCTGTCGGTAATA | Designed for Current Study |
| 28S | GGCGAAGCCAGAGGAAACT | GACGACCGATTTGCACGTC | Kaiser et al., 2000 Microbiology, 146:3217-3226. |
